# Comparison of two different combined test strips with fluorescent microspheres or colored microspheres as tracers for rotavirus and adenovirus detection

**DOI:** 10.1186/s12985-018-0951-5

**Published:** 2018-03-13

**Authors:** Na Jiang, Lei Shi, Jieping Lin, Lifang Zhang, Yanxia Peng, Huiying Sheng, Ping Wu, Qingjun Pan

**Affiliations:** 1 Clinical Research Center, Institute of Nephrology, Affiliated Hospital of Guangdong Medical University, Zhanjiang, Guangdong China; 2 Division of Endocrinology and Metabolism, Guangzhou Women and Children’s Medical Centre, Guangzhou, Guangdong China

**Keywords:** Rotavirus, Adenovirus, Fluorescent microspheres, Colored microspheres, Sensitivity, Specificity

## Abstract

**Background:**

Rotavirus (RV) and enteric adenovirus (AdV) mainly cause infantile infectious gastroenteritis. Several separate test methods for the detection of RV or AdV are currently available, but few tests are able to simultaneously detect both RV and AdV viruses, especially in primary medical institutions.

**Methods:**

The present study was mainly designed to compare the performance of two combined test strips for the detection of RV and AdV: a rotavirus–adenovirus strip with fluorescent microspheres for tracers (FMT); and the CerTest rotavirus–adenovirus blister strip with colored microspheres for tracers (CMT). To test the strips cultures of RV, AdV and from other enteric pathogens were used, in addition to 350 stool specimens from 45 symptomatic patients with gastrointestinal infections.

**Results:**

Detection thresholds for RV and AdV cultures using serial dilutions showed that the sensitivity of FMT was significantly higher than that of CMT (both *P* < 0.05). Specificity evaluation demonstrated that with culture mixtures of Coxsackie (A16), ECHO (type30), and entero- (EV71) viruses there was no detection of cross reaction using the two test strips, i.e., all the results were negative. With regard to the detection of RV in 350 clinical specimens, the total coincidence rate was 92.9%, the positive coincidence rate was 98.2%, and the negative coincidence rate was 90.8%. With regard to AdV detection, the total coincidence rate was 95.4%, the positive coincidence rate was 95.2%, and the negative coincidence rate was 95.5%.

**Conclusions:**

FMT performed better than CMT with regard to the combined detection of RV and AdV.

## Background

Rotavirus (RV) and enteric adenovirus (AdV) infections mainly result in acute infantile gastroenteritis, transmitted via the fecal–oral route. The symptoms include watery diarrhea, vomiting, headaches, fever, and gastralgic abdominal spasms [1, 2]. Symptoms may appear 1–2 days after viral infection, and persist for 1–10 days after the development of gastroenteritis, but different viral infections have different durations (RV approximately 3 days; AdV 5–8 days) [3], and the clinical features differ. Therefore, differential diagnosis is very important, especially for early diagnosis and treatment. Symptoms may also manifest in adults [4, 5]. RV and AdV account for the first and second most common viral infections among children under 5 years, respectively [6–8]. Therefore, RV and AdV are tested for simultaneously during the clinical diagnosis of viral gastroenteritis [4, 5].

Several methods for detecting RV and AdV are currently available: 1) viruses were cultured in cells, and immunological or molecular biological methods are used to identify the viruses [9]; 2) immunological methods are used directly to detect the presence of antigens or IgM and IgG antibodies in the specimens [10–12]; and 3) molecular biological methods are used directly to detect antigens [10, 13]. Methods 1 and 3 are limited with respect to technology and equipment, especially in primary medical institutions and field tests. With regard to Method 2, tests based on gold immunochromatographic assays, in which colloidal gold is used as the tracer utilized in antigen–antibody reactions, are simple, fast, require no special equipment or professionals, and have been used extensively in several fields including medicine and food safety [14–16]. However, this method has low sensitivity [17], and may fail to detect low titers of RV and AdV.

An emerging detection technique uses fluorescent microspheres [18, 19] instead of colloidal gold particles [20]. Reaction bands can be observed with the naked eye following illumination with an ordinary UV lamp or special equipment. This technique is comparatively sensitive and stable [21, 22]. High-resolution spectra that are recognizable by the naked eye can be obtained from stimulated microspheres, with sensitivities higher than those of colloidal gold particles [23].The relevant products are currently at the basic research and development stage, and are based on fluorescent microspheres (mainly for single-item detection) [24–26]. Colored microspheres are also used as detection tracers. They can be produced in any color (e.g., black, blue, orange, gray, red, pink, green, violet, or yellow) and in various sizes (most from 10 μm to 1000 μm) from materials including polyethylene, silica, and glass [27, 28]. However, the sensitivity of colored microsphere tracers is not well known, and will be evaluated in the present study.

The objective of the present study was to compare the two different methods—using fluorescent microspheretracers (FMT) or colored microspheretracers (CMT)—and to demonstrate the efficiency of FMT in relation to CMT in combined RV–AdV detection. This will provide a more reliable mean of detecting RV and AdV infections, and will have a significant clinical effect on the proper diagnosis and therapy of viral gastrointestinal infections, especially in primary medical institutions.

## Materials and methods

### Viruses

Viruses RV (Wa strain), AdV (type 40), Coxsackie (A16), ECHO 116 (type 30), and entero- (EV71) viruses were multiplied in cell cultures with MA-104 cells, Hela cells, Hep-2 cells, RD cells and Vero cells using DMEM (Dulbecco’s Modified Eagle Medium) culture media or RPMI culture media with 10% Fetal Bovine Serum (FBS), respectively, and stored in the laboratory.

### Clinical samples

Clinical specimens were collected from 350 symptomatic patients with viral gastrointestinal infections from the in- and out-patient wards at the Affiliated Hospital of Guangdong Medical University, and from the Guangzhou Women and Children Medical Care Center between March 2014 and February 2016.The study participants included 214 males and 136 females of mean age 16.2 ± 14.7 years. All the patients provided written informed consent to participate. The study was approved by the Ethics Committee of the Affiliated Hospital of Guangdong Medical University (LL20140317486).

All the authors had access to information that could identify individual participants during and after data collection.

### Combined rotavirus–adenovirustest strips

**Fig. 1:**
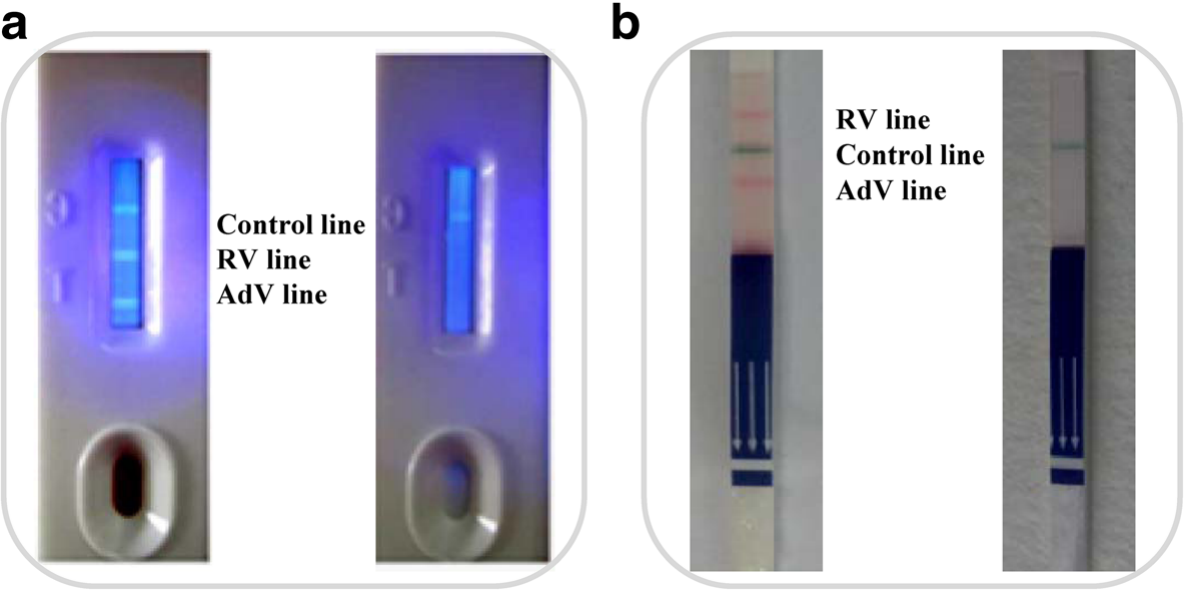
Test modes of the two combined detection test strips. Fluorescent microspheres for tracers (FMT): Control line (C); Detection line (T) including the enteric adenovirus (AdV) line (near the sample loading well) and the rotavirus (RV) line. Interpretation of results: after the test procedures, double positive (left) and negative (right) results were detected. Colored microspheres for tracers (CMT): Control line (blue); Detection line (red) including the AdV line (near the sample loading end) and the RV line. Interpretation of results: after the test procedures, double positive (left) and negative (right) results were detected

The combined rotavirus–adenovirus fluorescent microspheres test strip (FMT) was provided by Guangzhou Ruimu Biotechnology Co., Ltd. (Guangzhou, China). The tracers were fluorescent microspheres (phenylethene luminative monomeric copolymers; approximate diameter 100 nm) with excitation and emission wavelengths of 365 nm and 485 nm, respectively (Zhuhai Yongru Co., Ltd., Zhuhai, China). The detection process was performed according to the manufacturer’s instructions, and the test mode is shown in Fig. 1A.

A CerTest rotavirus–adenovirus blister test strip (CMT) was provided by CerTest Biotec S.L. (Zaragoza, Spain). The tracer is a colored microsphere (red for RV and AdV lines; blue for the control line). The detection process was performed according to the manufacturer’s instructions, and the test mode is shown in Fig. 1B.

### Rotavirus and adenovirus real-time reverse transcription polymerase chain reaction (real-time RT-PCR) kits

Rotavirus and adenovirus real-time RT-PCR detection kits were purchased from Guangdong Huayin Pharmaceutical Technology Co., Ltd. (Guangzhou, China).

### Sensitivity evaluation

RV and AdV were diluted and tested using the two strips. The initial concentrations of RV and AdV were 3.6 × 10^5^ plaque-forming units (PFU)/mL and 1.4 × 10^5^ PFU/mL, respectively. Three drops (150 μL each) of the viral culture mixture were simultaneously applied to the sample loading well of the test strips. The results were visible within 10 min.

### Evaluation of specificity

For specificity evaluation cultures of Coxsackie (A16), ECHO (type 30), and entero- 157 (EV71) viruses were used.

### Preparation of clinical samples

Clean bamboo sticks were used to efficiently mix and liquefy the fresh stool specimens comprising mucus, pus, and blood, etc. A thin tube was used to aspirate three drops (150 μL each) of the mixture, which was simultaneously applied to each of the test cards.

### Statistical analyses

The total coincidence rate, the positive coincidence rate, and the negative coincidence rate of CMT and FMT were calculated to evaluate detection in the clinical specimens [29]. Statistical analysis was subjected to a Student’s t test. *P* < 0.05 was considered statistically significant. Statistical analysis was performed with SPSS 15.0.

## Results

### Sensitivity evaluation of CMT and FMT

After two-fold serial dilutions of the RV cultures to 1/2^7^ (28 × 10^2^ PFU/mL), the CMT results were negative, whereas viruses were still detectable with FMT, even the dilutions to 1/2^8^ (14 × 10^2^ PFU/mL), the FMT results were still positive; After two-fold serial dilutions of the AdV cultures to 1/2^8^ (5.4 × 10^2^ PFU/mL), the CMT results were negative, whereas viruses were still detectable with FMT, even the dilutions to 1/2^9^ (2.7 × 10^2^ PFU/mL), the CMT results were still positive. Statistical analysis showed the sensitivity of FMT was significantly higher than that of CMT (both *P* < 0.05) (Table 1).

**Table 1:** Comparison of the two test strips results at two-fold serial dilutions of the RV and AdV cell cultured samples

Groups Methods		Two-fold serial dilutions									
		1/2	1/2^2^	1/2^3^	1/2^4^	1/2^5^	1/2^6^	1/2^7^	1/2^8^	1/2^9^	1/2^10^
RV	CMT	+	+	+	+	+	+	–	–	–	–
	FMT	+	+	+	+	+	+	+	+	–	–
AdV	CMT	+	+	+	+	+	+	+	–	–	–
	FMT	+	+	+	+	+	+	+	+	+	–

### Specificity evaluation of CMT and FMT

The detection results from the two test strips (Table 2) showed that the culture mixture comprising Coxsackie (A16), ECHO (type30), and entero- (EV71) viruses had no cross reaction, i.e., all the results were negative.

**Table 2:** Comparison of the test results with other viruses

Methods	Coxsackie virus (A16)	ECHO virus (type 30)	Enterovirus (EV71)
CMT	–	–	–
FMT	–	–	–

### Detection of RV and AdV in clinical specimens using CMT and FMT

Both strips were used to test 350 specimens, and the results are shown in Tables 3 and 4.

**Table 3:** Comparison of the test results of RV clinical specimens with the two test strips

FMT	CMT		Total
	Positive	Negative	
Positive	109	22	131
Negative	2	216	219
Total	111	239	350

**Table 4:** Comparison of the test results of AdV clinical specimens with the two test strips

FMT	CMT		Total
	Positive	Negative	
Positive	59	13	72
Negative	3	275	278
Total	62	288	350

For RV detection, the total coincidence rate of CMT and FMT was 92.9% [(109 + 216)/350)]. Compared to CMT, the positive coincidence rate of FMT was 98.2% [109/(109 + 2)], and the negative coincidence rate of FMT was 90.8% [216/(216 + 22)]. For AdV detection, the total coincidence rate of CMT and FMT was 95.4% [(59 + 275)/350)]. Compared to CMT, the positive coincidence rate of FMT was 95.2% [59/(59 + 3)], and the negative coincidence rate of FMT was 95.5% [275/(275 + 13)].

In addition to the two test strips, a rotavirus real-time RT-PCR detection kit was also used to detect RV in the clinical specimens [30]. The results showed that 20 of the 22 specimens that tested negative with CMT but positive with FMT, tested positive using the real-time RT-PCR detection method, and neither of the two specimens that had previously tested positive with CMT but negative with FMT gave a positive result.

Furthermore, in addition to the two test strips, an adenovirus real-time RT-PCR detection kit was also used to detect AdV in the clinical specimens [31]. The results showed that of the 13 specimens that had previously tested negative with CMT but were positive using FMT, 11 tested positive with real-time RT-PCR, and none of the three specimens that had previously tested positive with CMT but negative with FMT tested positive using real-time RT-PCR.

## Discussion

The use of colloidal gold test strips for the detection of RV and AdV is gradually increasing, both in China and abroad. However, owing to technological limitations, there are very few combined detection products. The CerTest rotavirus–adenovirus blister test strip (CMT) is popular in clinical practice and scientific research [32, 33]. However, CMT strips do sometimes fail to detect viruses (false negative results) during clinical tests owing to deficiencies in the colored microsphere technique, especially when there are insufficient quantities of RV and AdV in the stool specimens. Thus, there is a huge demand for lateral flow immunochromatographic techniques with highly sensitive fluorescent microspheres as tracers. Currently, there are few manufacturers of combined RV–AdV fluorescent microspheredetection test strips in China and abroad.

In the present study, CMT and real-time RT-PCR were used as the reference products to evaluate the detection performance of combined RV–AdV fluorescent microsphere detection test strips. CMT uses colored microspheres (red and blue, etc.) as the tracers, which produce significant results when used to detect diagnostic reagents in vitro. The efficiency of the test trips can them be determined. Thus, colored latex microsphere immunoassays have gradually attracted attention in the industry [34–36].

RV is the most common cause of severe acute gastroenteritis in children [1], and the most common enteropathogens have been extensively studied in China [37–39]. In the present study, when a combined RV–AdV FMT strip was used to detect viruses in the clinical specimens, the rate of positive RV detection in the 350 clinical specimens was 37.4% (131/350), and the rate of positive AdV detection was 20.6% (72/350). Such high infection rates demonstrate that it is necessary to detect both RV and AdV in clinical specimens from patients with gastroenteritis in China.

The results show that the combined RV–AdV FMT detection strips were significantly more sensitive than the CMT strips. Although the FMT technique seems to be more sensitive, it has some limitations that must be considered, such as the risk of false positives. Therefore, caution should be exercised, especially during the detection of weakly positive specimens [40]. Low specimen numbers also constitute a limitation. Hence, there is a requirement for further development by medical institutions, and for large-scale clinical specimen verification. There was cross reaction with the other common viral intestinal infections with FMT. The test results should be obtained within 30 min of specimen collection to provide timely, accurate, and reliable diagnoses in clinical settings.

## Conclusions

Both FMT and CMT were able to detect RV and AdV with high specificity, although FMT had higher detection sensitivity than CMT.

## Abbreviations

AdV: adenovirus
CMT: colored microsphere as the tracer
FMT: fluorescent microsphere
PFU: plaque forming unit
Real-Time RT-PCR: Real-Time Reverse Transcription-PCR
RV: Rotavirus
